# Prevention of Shiga toxin 1-caused colon injury by plant-derived recombinant IgA

**DOI:** 10.1038/s41598-022-22851-4

**Published:** 2022-10-26

**Authors:** Katsuhiro Nakanishi, Taichi Takase, Yuya Ohira, Ryota Ida, Noriko Mogi, Yuki Kikuchi, Minami Matsuda, Kohta Kurohane, Yoshihiro Akimoto, Junri Hayakawa, Hayato Kawakami, Yasuo Niwa, Hirokazu Kobayashi, Eiji Umemoto, Yasuyuki Imai

**Affiliations:** 1 Laboratory of Microbiology and Immunology, School of Pharmaceutical Sciences, University of Shizuoka, Shizuoka City, Shizuoka 422-8526 Japan; 2 Department of Anatomy, Kyorin University School of Medicine, Mitaka, Tokyo, 181-8611 Japan; 3 Laboratory for Electron Microscopy, Kyorin University School of Medicine, Mitaka, Tokyo, 181-8611 Japan; 4 Laboratory of Plant Molecular Improvement, Graduate Division of Nutritional and Environmental Sciences, University of Shizuoka, Shizuoka City, Shizuoka 422-8526 Japan

**Keywords:** Biotechnology, Immunology, Microbiology, Plant sciences, Diseases, Gastroenterology, Medical research, Pathogenesis

## Abstract

Immunoglobulin A (IgA) is a candidate antibody for oral passive immunization against mucosal pathogens like Shiga toxin-producing *Escherichia coli* (STEC). We previously established a mouse IgG monoclonal antibody (mAb) neutralizing Shiga toxin 1 (Stx1), a bacterial toxin secreted by STEC. We designed cDNA encoding an anti-Stx1 antibody, in which variable regions were from the IgG mAb and all domains of the heavy chain constant region from a mouse IgA mAb. Considering oral administration, we expressed the cDNA in a plant expression system aiming at the production of enough IgA at low cost. The recombinant-IgA expressed in *Arabidopsis thaliana* formed the dimeric IgA, bound to the B subunit of Stx1, and neutralized Stx1 toxicity to Vero cells. Colon injury was examined by exposing BALB/c mice to Stx1 via the intrarectal route. Epithelial cell death, loss of crypt and goblet cells from the distal colon were observed by electron microscopy. A loss of secretory granules containing MUC2 mucin and activation of caspase-3 were observed by immunohistochemical methods. Pretreatment of Stx1 with the plant-based recombinant IgA completely suppressed caspase-3 activation and loss of secretory granules. The results indicate that a plant-based recombinant IgA prevented colon damage caused by Stx1 in vivo.

## Introduction

The gastrointestinal (GI) tract is continuously exposed to foreign antigens including pathogenic microorganisms via the oral and nasal routes. Therefore, the GI tract is considered one of the main invasive sites for pathogens. To protect the body from infectious pathogens, Immunoglobulin A (IgA) antibodies are believed to act on the mucosal surface like in the GI tract^1^. It is expected that GI infections can be prevented if a sufficient amount of pathogen-specific IgA is artificially provided on the mucosal surface of the GI tract. There are two strategies for increasing IgA antibodies on the GI tract surface; mucosal vaccination and oral passive immunization with preformed IgA antibodies^2^. A mucosal vaccine can induce long-term IgA production, but several weeks are required for the induction of IgA after vaccination. On the other hand, oral passive immunization can work immediately after IgA administration but the effective time is shorter than that with a vaccine. Thus, IgA antibodies will be useful to treat acute infectious diseases including food poisoning based on oral passive immunity.

Shiga toxins (Stx) are bacterial toxins known as two immunologically distinctive types, Stx1 and Stx2^3^. Stx1 is produced by *Shigella dysenteriae* and by some pathogenic *Escherichia coli* strains that cause food poisoning^4^. The latter group is collectively called Shiga toxin-producing *E. coli* (STEC) that includes enterohemorrhagic *E. coli* (EHEC). Stx1 is secreted from these bacteria into the colon lumen, and it reaches the systemic circulation beyond the colonic epithelial barrier. On the other hand, Stx2 is produced only by STEC. A subset of patients infected with STEC develops into serious complications such as hemolytic uremic syndrome (HUS). In humans, it has been suggested that the development of HUS is more likely to occur when the lesion of STEC-related colitis is in the distal part of the colon^5^. HUS is more strongly associated with Stx2 production compared with Stx1^3^, but some cases of *S. dysenteriae* infection were reported to develop into HUS^4^. Mice are known to be resistant to severe diseases upon STEC infection. A mouse model involving direct inoculation of STEC strains into the stomach of weaned BALB/c mice^6^ and that involving repeated intraperitoneal injection of Stx2 into C57BL/6 mice^7^ were developed. Both models suggest the role of Stx2 in the severe systemic conditions. A recent report also suggests the importance of Stx2 in the development of systemic illness using streptomycin-treated BALB/c mice upon intragastric administration of streptomycin-resistant O26:H11 strains^8^.

Apart from the development of HUS and severe systemic diseases, it is still not fully understood how Stx initially reaches blood circulation^3^. In our hands, we previously found a restricted distribution of Stx1-binding sites on the distal colon epithelium of mice using a recombinant binding subunit of Stx1 (Stx1B)^9^. Furthermore, we showed that glycolipid ligands for Stx1B were selectively distributed in the distal colon. We also demonstrated that epithelial cells isolated from the distal colon, but not from the proximal colon, were susceptible to Stx1-induced apoptosis^10^. Thus, inhibition of Stx1-caused damage to the distal colon is expected to be important in the prevention of the aggravation of Stx1-caused illness in both humans and mice.

Stx1 is classified as an AB_5_ toxin that comprises one cytotoxic A-subunit and five cell-binding B-subunits (Stx1B)^11^. Binding to and internalization of Stx1B into the target cell is the first step of Stx1-induced cell death. Thus, inhibition of the binding of Stx1B to globotriaosylceramide (Gb3) or other cell surface receptors will prevent Stx1-induced cytotoxicity.

To achieve oral passive immunity against Stx1, we previously established a recombinant IgA-like antibody against Stx1B (hyIgA) by genetic engineering using an Stx1B-specific mouse IgG1 monoclonal antibody (mAb) (D11C6) and an Stx1B-specific mouse IgA mAb (G2G7)^12–14^. The heavy chain of the hyIgA consists of the N-terminus to the hinge region derived from IgG1 clone D11C6 and the Fc region from IgA clone G2G7^14^. The light chain is derived from D11C6. We are studying plant expression systems to produce recombinant antibodies, which are called plantibodies. We also established transgenic plants expressing hyIgA because a plant expression system has advantageous features for in vivo applications, such as cost-effectiveness in production and the minimal formulation required for mucosal administration^15–17^. The plant-derived hyIgA was shown to neutralize the cytotoxicity of Stx1 in vitro but the yield of hyIgA was not enough for use in experiments on Stx1 neutralization in vivo^18–21^.

In this study, we investigated the neutralization activity of Stx1-specific IgA in vivo. We first reorganized the hyIgA insert into a more effective one, in which all domains of the heavy chain constant region were from IgA. We showed that the new IgA molecule expressed in a transgenic plant neutralized Stx1 toxicity in vitro. We then developed a mouse model of distal colon injury by rectal administration of Stx1. By means of this model, we analyzed the neutralization activity of the Stx1-specific plant-based recombinant IgA.

## Results

### Production of transgenic plants expressing Stx1-neutralizing IgA antibody

**Figure 1:**
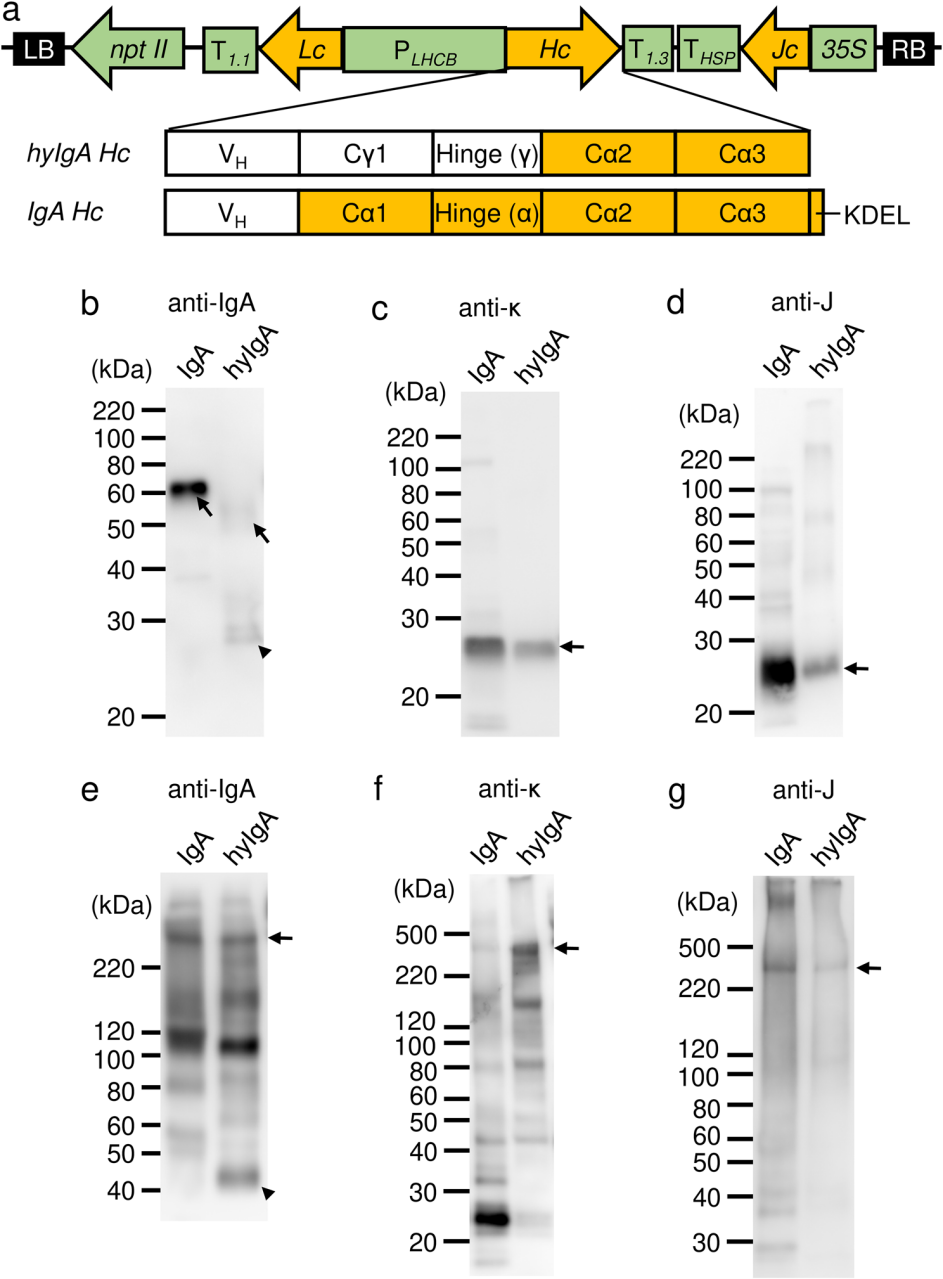
Production of IgA plantibody specific for Stx1B in a plant expression system. (**a**) Schematic diagram of the transfer-DNA (T-DNA) region of the Stx1B-specific dimeric IgA expression vector (pRI201/dimeric IgA). P_*LHCB*_, light-harvesting complex II (LHCB) promoter; T_*1.1*_ and T_*1.3*_, LHCB terminators; *Hc*, *IgA heavy chain* gene; *Lc*, *IgA light chain* gene; *Jc*, *immunoglobulin joining chain* gene; *35S*, cauliflower mosaic virus *35S* promoter; T_*HSP*_, terminator of *Arabidopsis heat shock protein 18.2* gene; *nptII*, *neomycin phosphotransferase II* gene; LB, left border of T-DNA region; RB, right border of T-DNA region. Immunoglobulin domain organizations of the heavy chain inserts for the hyIgA and IgA are shown in parallel. VH, variable region; Cγ, heavy chain constant regions from IgG1; Cα, those from IgA; Hinge (γ or α), hinge region from IgG1 or IgA; KDEL, the endoplasmic reticulum retention signal peptide. (**b–g**) Immunoblot analysis of leaf extracts of transgenic *Arabidopsis thaliana*. Leaf proteins (**b,c,e,f**, 10 ng/lane as IgA; **d,g**, 30 ng/lane as IgA) were separated by SDS-PAGE under reducing (**b–d**: 12% gel) and non-reducing (**e–g**: 4–15% gradient gel) conditions, and then blotted onto PVDF membranes. Each immunoglobulin chain was detected with anti-IgA heavy chain (**b,e**), anti-κ light chain (**c,f**), and anti-J chain (**d,g**) antibodies, respectively. Arrows indicate the intact immunoglobulin chains and arrowheads indicate the incomplete IgA heavy chains. Leaf extracts of Stx1B-specific IgA transgenic *A. thaliana* (IgA) or hyIgA transgenic *A. thaliana* (hyIgA) were analyzed.

To improve the expression level of hyIgA in transgenic plants (hyIgA-transgenic plants) previously established^18,19^, we modified hyIgA genes and constructed an expression vector capable of producing dimeric IgA (Fig. 1a). Previous studies suggested that the CH1 and hinge region of mouse IgG1 is vulnerable to digestion by plant intracellular proteases^19,22–24^. Thus, the mouse IgG-derived CH1 and hinge region sequence within the hyIgA heavy chain gene was replaced by that of mouse IgA to avoid digestion by plant proteases. Additionally, the codon usage of heavy, light and J chain genes was optimized for *Arabidopsis thaliana*, and the endoplasmic reticulum (ER) retention signal peptide (KDEL amino acid) was added to the C-terminal of each chain to increase the expression level in plant cells^25,26^. Using the resulting modified IgA expression vector, we produced transgenic *A. thaliana* plants expressing Stx1-specific IgA (IgA-transgenic plant) through *Agrobacterium*-mediated transformation.

The expression of IgA protein in a transgenic plant leaf extract was assessed by SDS-PAGE and immunoblot analysis (Fig. 1b–g). An extract derived from a transgenic plant expressing dimeric hyIgA^18^ was used for comparison. Under reducing conditions, H chain (arrow) was detected at 60 kDa in the IgA-transgenic plant sample (Fig. 1b). HyIgA-transgenic plants contained complete hyIgA-H chains (arrow) and an incomplete one (arrowhead), which was smaller than 30 kDa (Fig. 1b). L (Fig. 1c) and J (Fig. 1d) chains were clearly detected in the extract of IgA- and hyIgA-transgenic plants.

Under non-reducing conditions, both anti-IgA heavy chain and anti-J chain antibodies revealed an assembled IgA dimer at around 300 kDa in the extract of IgA- as well as hyIgA-transgenic plants (Fig. 1e,g, arrows). L chain was incorporated in the 300-kDa band obtained from the hyIgA- but not from the IgA-transgenic plant extract (Fig. 1f). The latter extract exhibited a strong band (25 kDa) reactive with anti-κ antibodies, suggesting the free light chain. This is consistent with the fact that the L chain associates with the H chain through a non-covalent interaction but not through a disulfide bond in the case of IgA of BALB/c mouse origin^27^. Collectively, the 300-kDa band of the IgA-transgenic plant contained four IgA heavy chains and one J chain but no L chain (dimeric IgA without L chains). Current immunoblot methods cannot determine whether the L chains are dissociated from dimeric IgA during processing for SDS-PAGE or such incomplete dimers are produced in the plant.

Other bands of lower molecular weights were also observed on IgA heavy chain detection (Fig. 1e). The 110 kDa band probably represents a dimer of the H chain. Lower molecular weight bands may represent a single heavy chain. The monomeric IgA (160 kDa) was observed in the hyIgA lane upon staining with anti-IgA heavy (Fig. 1e) and anti-κ (Fig. 1f) antibodies, but such a band was absent from the IgA lane. The incomplete H chain was observed as a dimer of over 40 kDa for hyIgA (Fig. 1e, arrowhead).

### Improvement of antigen recognition and neutralization activity of the Stx1-specific IgA plantibody

**Figure 2:**
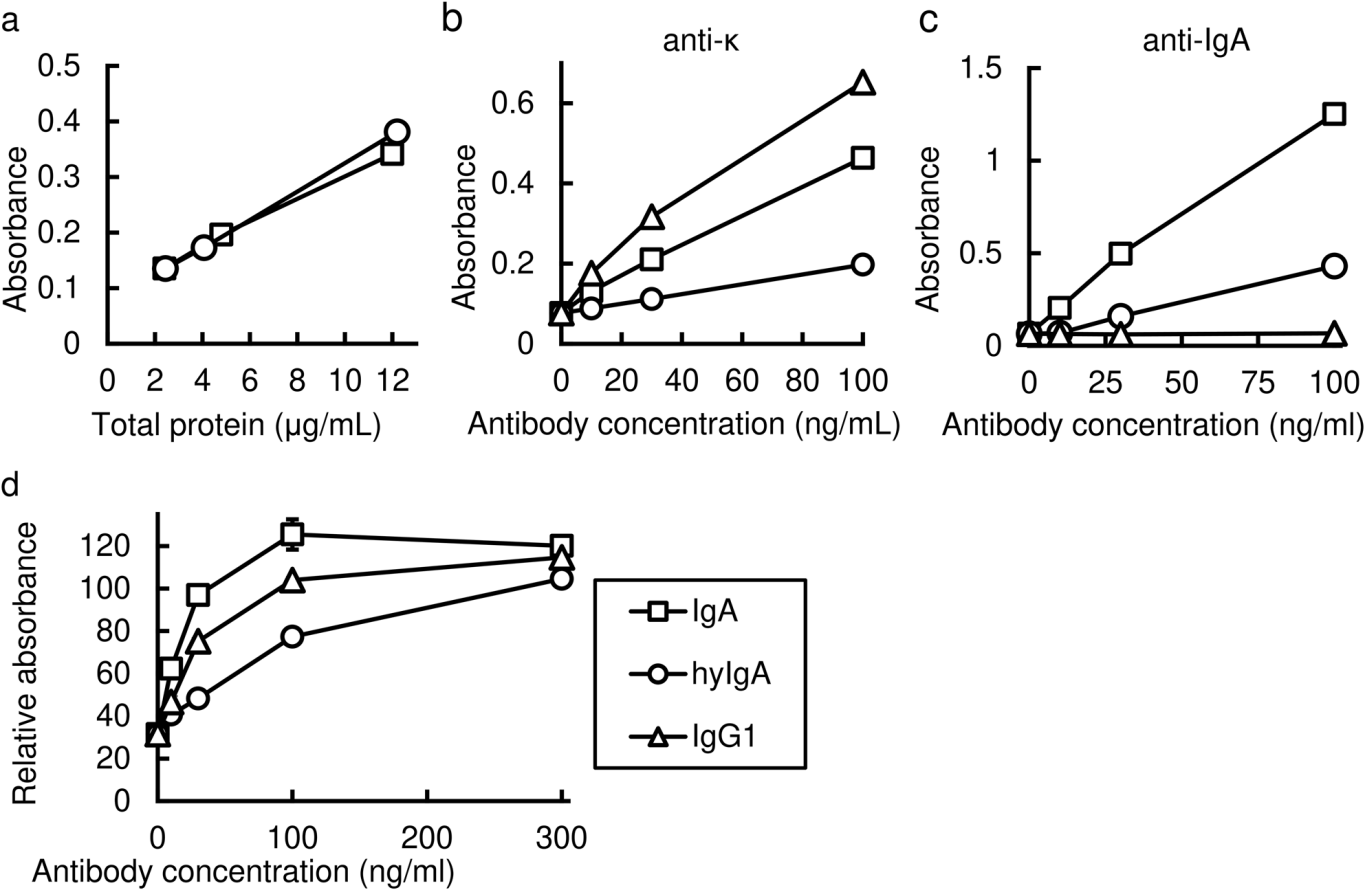
Antigen-recognition and neutralization activity of the Stx1B-specific IgA plantibody in vitro. (**a**) Detection of assembled IgA by sandwich ELISA. Signals for IgA carrying both heavy and light chains (ordinate) were plotted against the protein concentrations (abscissa) in the leaf extracts of transgenic plants that express IgA (squares) or hyIgA (circles). Data are expressed as means for triplicate determinations, and SDs were shorter than each symbol. (**b,c**) Dose-dependent binding of IgA and an IgG1 mAb antibody to the immobilized Stx1B in response to antibody concentration (abscissa). Signals for the bound antibodies were detected using anti-κ light chain antibodies (ordinate) (**b**) or anti-IgA heavy chain antibodies (**c**). Samples from IgA (squares) or hyIgA (circles) transgenic plants, or hybridoma-derived mouse IgG1 mAb (triangles) were analyzed. Data are expressed as means of duplicate determinations, and the ranges were shorter than each symbol. (**d**) Neutralization of Stx1 cytotoxicity by IgA plantibodies and the IgG1 mAb in vitro. One hundred pg/mL of Stx1 was treated with various concentrations of Stx1B-specific antibodies (abscissa) for 1 h. Vero cells were incubated with an Stx1/antibody mixture for 45 h, and then live cells were quantitated by the WST-8 assay. The ordinate indicates the relative OD450 for each culture compared with the control condition (no toxin exposure). The indicated amount of antibody (abscissa) present in the leaf extract of IgA- (squares) or hyIgA- (circles) transgenic plants, or in the IgG1-producing hybridoma culture supernatants (triangles) was added to each culture. Data are expressed as means ± SD of triplicate cell cultures. The results are representative of three experiments.

To make sure that IgA heavy chains are assembled with light chains, we developed a sandwich ELISA method, in which the anti-κ light chain was used to capture IgA and anti-α heavy chain for detection. As shown in Fig. 2a, samples from both IgA-transgenic and hyIgA-transgenic plants exhibited dose-dependent increases in signals representing the assembled IgA consisting of both H and L chains. These results clearly demonstrated the assembly of IgA H and L chains in the IgA-transgenic plants.

This ELISA system was utilized to quantify the assembled IgA protein in leaf extracts using mouse myeloma TEPC 15 proteins as IgA standards. The production level of modified IgA plantibodies was 17.2 ± 3.4 μg/g of leaf fresh weight (LFW) in three independent measurements (mean and SD). This value is similar to that for hyIgA in plants (18.8 ± 0.8 μg/g LFW).

We then compared the binding of IgA to immobilized Stx1B between the IgA-transgenic and hyIgA-transgenic plants by means of ELISA using anti-κ light chain antibodies (Fig. 2b). Because the binding activity of IgA was also compared with that of D11C6 mouse IgG1 mAb, anti-κ light chain antibodies were used as common detection antibodies. Dose-dependent signals representing antigen recognition were seen for IgA (squares), hyIgA (circles), and IgG1 (triangles). IgA was shown to bind to Stx1B more efficiently than hyIgA. The dose–response curve demonstrated that the signal representing the antigen recognition of IgA to the immobilized Stx1B was approximately fourfold higher than that for hyIgA. Such signal for IgG (triangles) to Stx1B was twofold higher than that for IgA (Fig. 2b). Superior antigen recognition activity of IgA over hyIgA was confirmed by using an anti-α detection antibody. A dose–response curve demonstrated that the signal representing the antigen recognition by IgA was approximately fivefold higher than that of hyIgA (Fig. 2c). The binding of IgG to the immobilized Stx1B was not detected by anti-α antibody, suggesting the IgA-specificity of this experiment. Determination of dissociation constant (Kd) is an important part of the molecular characterization of antibodies. However, it is not feasible to determine the Kd by means of ELISA methods, and it requires a large amount of purified plantibody samples for the determination by means of physicochemical methods, such as surface plasmon resonance assay. From these reasons, we did not measure the Kd between Stx1B and antibodies in this study. Further studies are needed to elucidate this issue.

The next question was whether the efficient antigen recognition by IgA reflects the toxin-neutralizing activity. A decrease in the live cell number upon exposure to Stx1 was examined by means of WST-8 assaying using Stx1-sensitive Vero cells (Fig. 2d). A 70% decrease in the live cell signal was observed with 100 pg/mL of Stx1 compared with the culture without toxin exposure. The loss of live cells was reversed when Stx1 was preincubated with IgA (squares), hyIgA (circles), or IgG1 (triangles). Thirty ng/mL of IgA completely neutralized the Stx1 toxicity, whereas 300 ng/mL of hyIgA was required for complete neutralization. In contrast, 100 ng/mL was required for the complete neutralization by IgG1 mAb. These results indicated that the improved recombinant IgA was functionally superior to the hyIgA and IgG mAb as to toxin neutralization in vitro.

### Intrarectal administration for Stx1-induced colon lesions in mice

**Figure 3:**
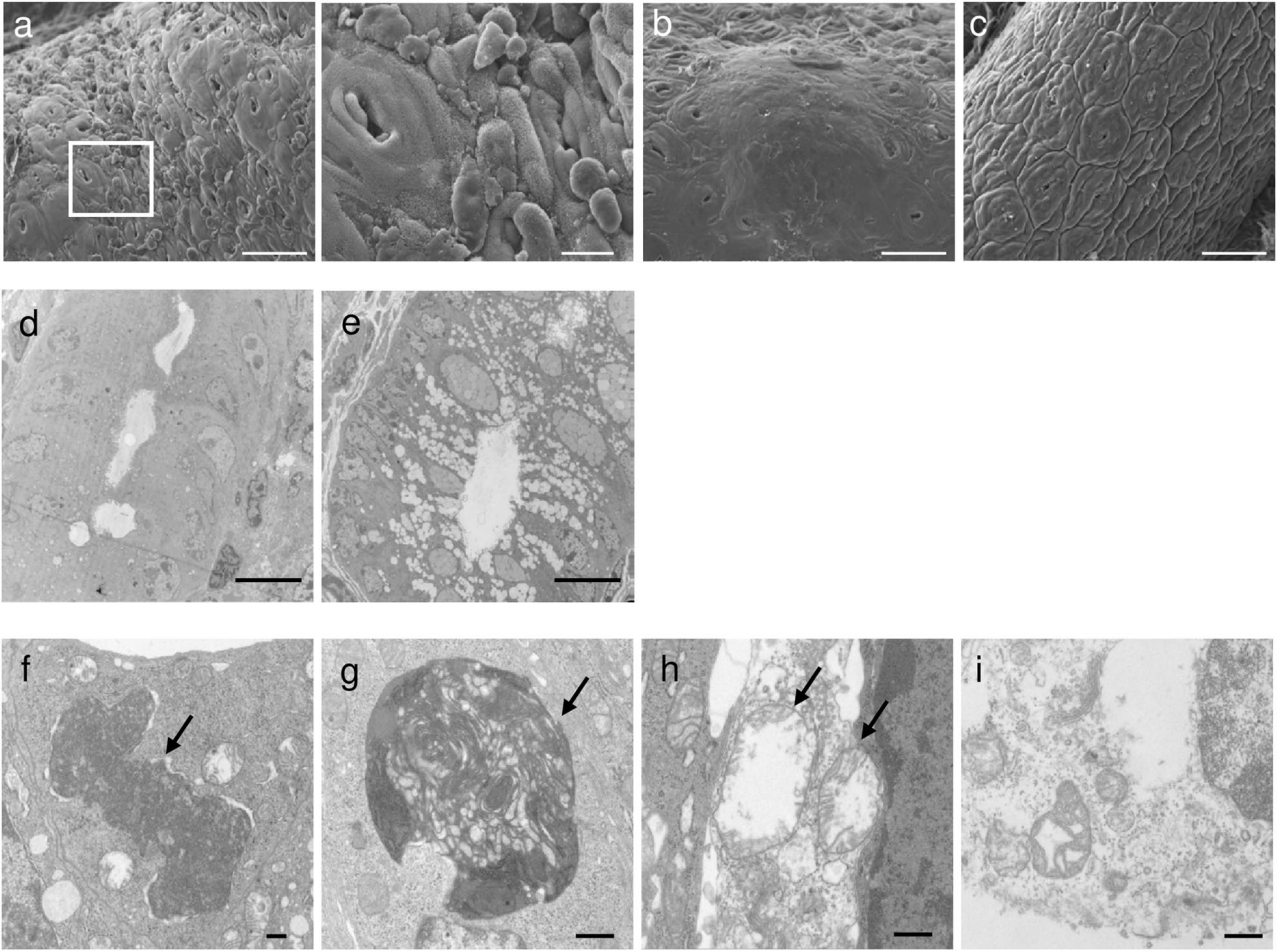
Electron-microscopic analysis of Stx1-exposed mouse colon epithelium. Mouse colon epithelium was exposed to 0.5 μg of Stx1 or PBS (buffer control) for 16 h by intrarectal administration. The Stx1-exposed colon tissue (**a,b,d,f–i**) and buffer control (**c,e**) were analyzed by SEM (**a–c**) and TEM (**d–i**). **(a)**-right shows a high magnification image of the white squared area in (**a)**-left. Arrows indicate features of cell death. Bars represent 50 μm (**a**-left**,b,c**), 10 μm (**a**-right**,d,e**), or 500 nm (**f–i**).

To assess the toxicity of Stx1 toward colon tissue and its prevention by the plantibody, we tried to induce colon lesions with Stx1 in vivo. We previously demonstrated that the binding sites for Stx1B were localized on the mucosal epithelium of the mouse distal colon^9^. Thus, we administered 0.5 μg of Stx1 intrarectally to expose colon epithelial cells to Stx1. We determined the morphological changes of the distal colon tissue by electron microscopy (Fig. 3).

On scanning electron microscopy (SEM), destruction of part of the mucosa with fragmented and peeled epithelia (Fig. 3a), and epithelial ridge formation on the mucosal surface and a loss of crypts from such areas (Fig. 3b) were observed in the Stx1-administered mice. These features were not observed in the Phosphate-buffered saline (PBS)-administered mice (Fig. 3c).

On transmission electron microscopy (TEM), decreases in the numbers of goblet cells and their secretory granules (Fig. 3d) were observed in the Stx1-treated colon tissue as compared with in the PBS control (Fig. 3e). Some features of both apoptotic and necrotic cell death were seen in the epithelial cells of the Stx1-treated colon tissue at high magnification (Fig. 3f–i). Shrinkage of nuclei (Fig. 3f, arrow) and uptake of organelles by lysosomes (Fig. 3g, arrow) were features of apoptosis. Swelling of mitochondria (Fig. 3h, arrow) and disruption of the cell membrane (Fig. 3i) suggested necrosis.

**Figure 4:**
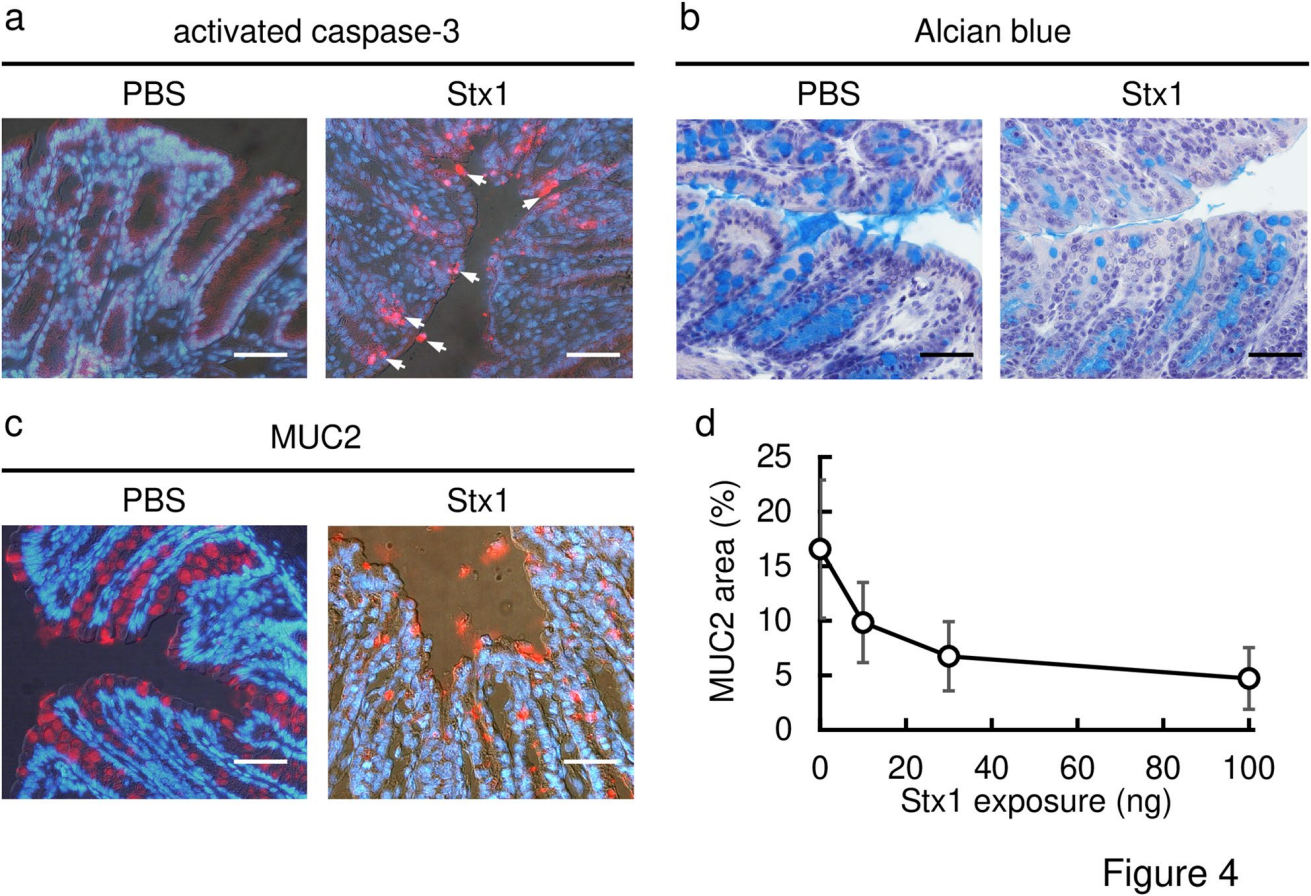
Activation of caspase-3 and depletion of secretory granules in the colon epithelium upon intrarectal exposure to Stx1. Frozen sections (**a,c**) or paraffin sections (**b**) of colon tissue from Stx1-exposed or control (PBS) mice were stained with anti-activated caspase-3 antibodies (red in **a**), Alcian blue dye (blue in **b**), or anti-MUC2 antibodies (red in **c**). Nuclei were visualized by staining with DAPI (blue in **a,c**), and with hematoxylin (violet in **b**). Arrows indicate signals from activated caspase-3 (**a**). Fixation of tissues was performed with formalin-PBS (**a**,**c**) or Carnoy’s fixative (**b**). Bars represent 50 μm. Each mouse was exposed to 100 ng of Stx1. (**d**) Quantification of secretory granules in the colon tissues in response to the amount of Stx1 (abscissa) exposed to each mouse. The ordinate indicates the percentage of the MUC2-positive area divided by the total area of the mucous epithelium (mean ± SEM) from four independent mice. There were no exclusions of data points.

Histological analyses were also performed using frozen (immunostaining) or paraffin (Alcian blue) sections (Fig. 4). When 100 ng of Stx1 was intrarectally administered, the activation of caspase-3 in the Stx1-treated colon epithelial cells was detected on immunostaining with activated caspase-3 specific antibodies (Fig. 4a, red signals). In the PBS-administered mouse colon, caspase-3 activation was not observed. For the detection of secretory granules, colon sections were stained with Alcian blue dye, which binds to acidic polysaccharides in secretory granules (Fig. 4b, stained blue). The density of Alcian blue staining was markedly decreased in the Stx1-exposed mouse distal colon crypts as compared with in the PBS-treated control mice. A similar result was obtained on immunostaining for MUC2, a major colonic mucin (Fig. 4c, red signals). A decrease in the MUC2-positive area in a colon tissue section was observed in an Stx1 concentration-dependent manner (Fig. 4d). These results indicated that Stx1 induces damage to colon epithelial cells in vivo via intrarectal exposure.

### The Stx1-specific IgA plantibody prevented colon injury induced by Stx1

**Figure 5:**
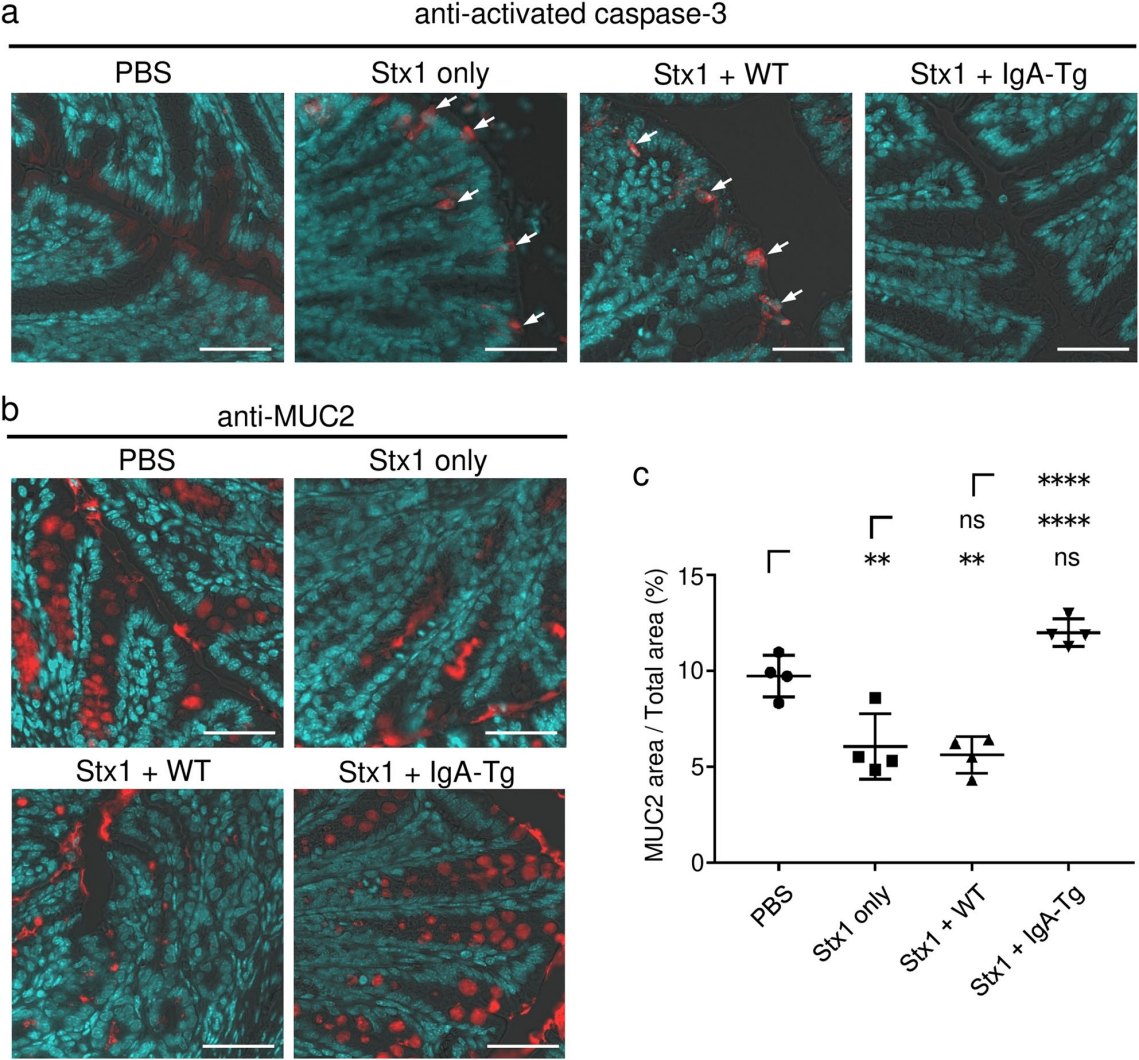
Prevention of Stx1-caused colon injury by an IgA plantibody specific for Stx1. Sixty ng of Stx1 was treated with the leaf extract of an IgA transgenic plant (Stx1 + IgA-Tg) containing 8 μg IgA (1.6 mg total proteins), that of a wild-type plant (Stx1 + WT) containing 1.6 mg of total protein, or PBS (indicated as Stx1 only) for 1 h. Mice were then exposed to each sample containing Stx1 or to PBS alone (indicated as PBS) through intrarectal administration. After 16 h, colon tissues were collected and embedded in O.C.T. compound. Frozen sections of the colon tissues were stained with anti-activated caspase-3 (**a**) or anti-MUC2 (**b,c**) antibodies. Nuclei were counterstained with DAPI (**a,b**). Arrows indicate signals from activated caspase-3 (**a**). Bars represent 50 μm. The percentages of the MUC2-positive area divided by the total area of the mucous epithelium are plotted (**c**). Horizontal bars represent the means for individual groups and error bars are SD (*n* = 4). There were no exclusions of data points. Statistical significance was analyzed by ANOVA followed by the Tukey test. **P < 0.01, ****P < 0.0001, ns *P* > 0.05.

Finally, we examined whether the Stx1-specific IgA prevents Stx1-induced colon injury in vivo (Fig. 5). Sixty ng of Stx1 per mouse was a sufficient dose to induce caspase-3 activation as well as a decrease of secretory granules in the mouse colon tissue (Fig. 5a,b; Stx1 only). Both signs corresponding to the Stx1-induced colon injury were inhibited when 60 ng of Stx1 had been pretreated with an IgA-transgenic leaf extract (Stx1 + IgA-Tg), which contained 1.6 mg protein and 8 μg of Stx1B-specific IgA (Fig. 5a,b). On calculation of the percentage of the MUC2-positive area (Fig. 5c), treatment with toxin alone (Stx1 only) gave a significantly lower value than that for the no toxin control (PBS). In contrast, the value for Stx1 + IgA-Tg conditions was comparable to that for a no toxin control, indicating complete recovery. The preventive effects were due to the IgA plantibody, not *A. thaliana*-derived proteins, because the wild-type plant (Stx1 + WT) extract, which included 1.6 mg of leaf protein, did not prevent caspase-3 activation (Fig. 5a) or the MUC2-positive area decrease (Fig. 5b,c).

These results demonstrated that a plant-based IgA could prevent in vivo colon damage through toxin neutralization.

## Discussion

IgA is a major molecule for protection from mucosal pathogens such as those involved in Stx1-caused food poisoning. An IgA mAb specific for a virulence factor of mucosal pathogens is expected to prevent infectious disease on application to the mucosal surface. To demonstrate the preventive effect of Stx1-secific IgA on the gut mucosal surface, we produced an anti-Stx1 IgA mAb by means of a plant expression system. We then evaluated the preventive effect using a mouse model of Stx1-induced colon injury in vivo.

Many strategies have been reported for increasing the yield of recombinant proteins in a plant expression system. These include optimal transgene expression at the transcription and translation levels, and enhancement of the stability of recombinant proteins^28^. To increase the production of IgA in transgenic plants, we modified hyIgA genes established in our earlier studies^14,18^ as to the following three points. Optimization of the codon usage of hyIgA genes for *A. thaliana*, addition of an ER-retention signal (KDEL) to the C-terminus, and replacement of the CH1 and hinge region of the H chain of mouse IgG1 with the IgA one. These modifications aimed at improving mRNA translation (codon optimization), increasing the folding and assembly of IgA (KDEL), and removing cleavage sites used by plant proteases (replacement of the CH1 and hinge regions)^18,22,25,26^. The apparent production level of IgA, which is based on the mass of proteins having both H and L chains per fresh leaf weight, was not increased by these modifications. However, functionally, the antigen recognition and neutralization activities of IgA increased by fourfold and tenfold compared with those of hyIgA, respectively, based on the assembled IgA concentrations (Fig. 2b,c). These results indicate that the modifications increased the recovery of functional plant-based IgA. However, it is natural to ask which modification is the basis for the increased functionality of IgA. Because we gave priority to the production of in vivo-effective IgA at a reasonable cost, we made as many modifications as we could think of at this time. Further studies are needed to determine which modifications are useful or not.

We calculated the production cost of IgA that can neutralize the toxicity caused by 1 μg of Stx1 in vitro based on live Vero cell measurement assaying (Fig. 2c). For the IgA-plantibody described in this report, it was calculated to be $13 in our laboratory. As to the hyIgA-plantibody, the cost was $33. In contrast, as to the hyIgA produced in Chinese Hamster Ovary cells^29^, the cost was $293 in our previous experiments. These figures indicated a practical advantage of the IgA-plantibody for testing the in vivo efficacy.

In the immunoblot analyses of IgA under reducing conditions, H, L and J chains were observed. Although H and J chains appeared to be incorporated to the IgA dimers, L chains were absent (Fig. 1f). Because the IgA gene was derived from BALB/c mice, the H and L chains are associated via a non-covalent interaction and not a disulfide bond^27^. In addition, the association between H and L chains in the IgA was substantiated by ELISA, in which anti-κ chain antibodies were used to capture IgA and anti-α antibodies for detection. This sandwich ELISA system detects an H–L chain complex even if the antigen-binding region of IgA is absent or inactive, because the antibodies used in the ELISA are directed to the constant region of L and H chains. It is conceivable that the presence of an inactive H–L chain complex resulted in the reduced antigen recognition and neutralization activities of hyIgA compared with those of IgA. The deletion of plant protease-sensitive sites by gene modification seemed to be effective for increasing the functional yield of IgA plantibody.

On immunoblot analysis using anti-IgA heavy chain antibodies under non-reducing conditions (Fig. 1e), the signal intensity of the dimeric IgA without L chains (300-kDa band), which contains four H and one J chain, was weaker than that of the H chain dimer (a band at 110 kDa), which contains two H chains. Although it has not been examined, one possibility is that the dimeric IgA is produced through the association of two monomeric IgA (consisting of two H and L chains) with a J chain in the plant, yielding a molecule consisting of four H, four L and one J chain (dimeric IgA). Processing for SDS-PAGE caused L chain dissociation from dimeric and monomeric IgA, yielding the dimeric IgA without L chains and the H chain dimer, respectively. It has been reported that the formation of a disulfide bond between the J chain and monomeric IgA is relatively inefficient compared with the downstream reaction, which produces a secretory IgA (S-IgA) in a transient plant expression system. Furthermore, it has been reported that a substantial quantity of monomeric IgA remained in plant cells even if the J chain was over-expressed in the plant ^30^. Thus, it is not surprising that a substantial amount of monomeric IgA remained in our case. The monomeric IgA might lose L chains on SDS-PAGE processing, then the H chain dimer would become abundant in the immunoblot (Fig. 1e). Thus, the improvement of the efficiency of binding of a J chain to monomeric IgA is an important challenge to produce SIgA in a plant expression system.

The effect of reformatting of IgG into IgA was examined as to humanized IgG mAbs, palivizumab and motavizumab, which are specific for respiratory syncytial virus. The efficacy was reported to be impaired after reformatting to IgA^31^. Despite of the title of the literature, their data indicated that the virus neutralization activity was only slightly reduced after reformatting to human IgA. In the present study, neutralizing activity against Stx1 did not decrease but rather increased after replacement of the CH1 and hinge regions of IgG1 with those of IgA. Our results demonstrated that the structural platform of IgA is not inferior as to toxin neutralization. This has also been shown through a comparison between the recombinant hyIgA expressed in CHO cells and the original IgG1 mAb D11C6^29^. It is still possible that the utility of IgA depends on the target antigens and the origin of the antibody genes.

We previously demonstrated that a recombinant Stx1B binds to the mucosal epithelium of the mouse distal colon, especially to the colonic crypts ^9^. We then demonstrated that epithelial cells isolated from mouse distal colon were susceptible to Stx1-induced apoptosis in vitro^10^. After 16-h exposure to Stx1, cells exhibiting DNA fragmentation, as revealed by TUNEL assaying, were observed under a microscope.

With these backgrounds, BALB/c mice were intrarectally exposed to Stx1 and the morphological changes in the distal colon epithelium were analyzed. On electron microscopic analysis, Stx1 was shown to induce epithelial cell death and crypt loss, to decrease the number of goblet cells and to deplete secretory granules. Signs of apoptosis (nuclear shrinkage, autophagy of organelles) as well as ones of necrosis (mitochondria swelling, cell membrane disruption) were observed (Fig. 3). We do not know whether the signs of necrosis or those associated with other types of programmed cell death (Fig. 3h,i) would be observed with exposure to a similar dose of Stx1. It is not known whether those changes occur following a similar timeline after exposure. Answers for these questions will be informative to understand the mechanism of Stx1 toxicity. Because we focused on whether the anti-Stx1 IgA plantibody prevents colon injury induced by Stx1, we focused on apoptosis and secretory granule losses at this time. Signs of colon injury, such as caspase-3 activation (a sign of apoptosis) and decreases in the numbers of goblet cells and secretory granules, were also observed with histochemical methods (Fig. 4). It should be noted that colon epithelial lesions have also been reported upon infection by Stx-producing bacteria in mice, rabbits, monkeys and humans^32–38^. It is conceivable that other virulence factors of bacteria play roles in the pathogenesis of colon injury. However, our results suggested that Stx1 is directly involved in the initial injury to the colon epithelium, at least in part.

As to the severe systemic conditions such as HUS, a central role of Stx2 was experimentally demonstrated by means of mouse models recently^8^. Streptomycin (Str)-treated BALB/c mice were orally infected with an Str-resistant STEC strain (O26:H11) producing both Stx1a and Stx2a, and the Stx-production was induced by intraperitoneal (ip) injection of Ciprofloxacin (Cip). Intravenous (iv) injection of an anti-Stx2 mAb protected animals from death but an anti-Stx1 mAb did not. Second, when mice were infected without Cip-treatment, iv-injection of a monoclonal or polyclonal anti-Stx1 antibodies aggravated the loss of weight while an anti-Stx2 mAb did not. Third, the B subunit of Stx1a was intraperitoneally injected into mice at 12 h before intoxication by ip-injection of Stx2a. A delay of time to death was observed. Mainly from these results, the authors hypothesized that Stx1a may reduce the toxicity of Stx2a at the target organ level such as kidneys. It should be noted that colonic epithelial damage caused by Stx1 could lead to an increased translocation of Stx2 into systemic circulation. Alternatively, Stx2 may breach the colonic barrier by itself. Further experiments will be needed to address this issue.

To demonstrate the in vivo effect of the plant-based IgA, we adopted caspase-3 activation and MUC-2 release from goblet cells in the mouse distal colon as markers of colon injury. When Stx1 was pretreated with the plant-based IgA before intrarectal administration, caspse-3 activation and MUC-2 release were inhibited (Fig. 5). These results indicated that the Stx1-specific plant-based IgA could protect mice from colon injury on the mucosal surface. It could be excessive that 8 μg plant-based IgA was used against 60 ng Stx1 challenge (Fig. 5). This is 133-fold excess of IgA compared with Stx1 on a weight basis. Due to the limitation in the ability of plantibody production in our laboratory at present, we did not carry out dose response experiments. However, in vitro neutralization assays also indicated that a relatively large amount of IgA was required for complete toxin neutralization. Thus, 30 ng/mL plant-based IgA was required to inhibit Stx1 (100 pg/mL) toxicity completely (Fig. 2c). This is a 300-fold excess. Although further dose-dependent in vivo experiments will strengthen the efficacy of plant-based IgA, we think we performed in vivo experiments within an appropriate IgA versus Stx1 ratio range.

It has been proposed that Stx not only induces cell death but also enhances STEC adhesion to the epithelial surface^39,40^. Due to the inhibition of the receptor-toxin interaction, Stx1-specific IgA may also inhibit STEC colonization in the colon indirectly.

Further in vitro experiments on the toxin neutralization by IgA are needed in relation to the morphological changes observed in the colon cells after in vivo exposure to Stx1 (Fig. 3). We showed that the plant-based IgA effectively neutralizes Stx1 toxicity based on the live cell measurement assay results using Vero cells (Fig. 2c). It is feasible to test apoptosis inhibition using Vero cells, for example. However, we focused on the in vivo effect in the present study, demonstrating apoptosis inhibition in an in vivo model (Fig. 5a). Regarding the mechanism of toxin neutralization of cellular level, one may consider the use of human colon cell lines^19^ in terms of clinical relevance.

As to several mucosal pathogens, it has been reported that pathogen-specific IgA can prevent infection^31,41–45^. As to Stx, previous studies indicated that mucosal vaccination could prevent disease and induce production of toxin-specific IgA antibodies, however, the effect of passively administered Stx-specific IgA was not clarified^33,39,46^. To examine the therapeutic effects of the plant-based IgA on oral administration, a sufficient amount of plantibody needs to be produced. One solution is the use of plants with a higher biomass. We have already demonstrated that leaf lettuce (*Lactuca sativa*) can produce Stx1B-specific hyIgA^21^. Another consideration for successful oral administration of IgA is protease resistance. The importance of the secretory component (SC) for the protease resistance of SIgA has been proposed^47^. We have also produced a plant-derived SC, and demonstrated that it makes dimeric hyIgA resistant to pepsin and trypsin^20,48^. Thus, SIgA production in leaf lettuce is a practical solution for assessing the therapeutic effect of orally administered IgA in vivo.

Therapeutic antibodies applicable to the mucosal surface other than that of the intestine should also be highlighted. For example, the upper respiratory tract mucosa is a candidate site for the use of IgA plantibodies. For example, variable regions of IgG antibodies specific for the spike protein of SARS-CoV-2 can be utilized in our plant expression system for intranasal administration.

In conclusion, we established a transgenic plant expressing Stx1-specific IgA with toxin neutralizing activity. The plant-derived IgA could prevent Stx1-induced colon injury in mice. Our future goal is the development of oral passive immunity using IgA mAbs.

## Materials and methods

### Reagents

Stx1 holotoxin and a recombinant Stx1B were prepared as described previously^13,49^; cloning vectors pGEM5zf/T_*LHCB1.1*_, pGEM5zf/T_*LHCB1.3*_ and pGEM5zf/P_*LHCB*_ as described previously^18^; *Agrobacterium tumefaciens* (*Rhizobium radiobacter*) strain GV3101 as described previously^50^; and Stx1B-specific IgG1 mAb (D11C6) as described previously^13,29^. *A. thaliana* ecotype Col-0 was obtained from Arabidopsis Biological Resource Center (Columbus, OH, USA). Restriction enzymes *Nde*I, *Sac*I-HF, *Not*I-HF, *Sac*II and *Nsi*I-HF were purchased from New England BioLabs (Ipswich, MA, USA); *Sma*I, *Kpn*I, *Sal*I, binary vector pRI201-AN, Klenow fragment, a DNA Ligation Kit <LONG> and a DNA Ligation Kit <Mighty Mix> from Takara Bio (Shiga, Japan); *Hin*dIII from Nippon Gene (Tokyo, Japan); a protease inhibitor cocktail for plant cell and tissue extracts, myeloma proteins TEPC 15 (mouse IgA, κ) and MOPC 21 (mouse IgG1, κ), and Alcian Blue 8GX from Sigma-Aldrich (St. Louis, MO, USA); 2,2'-Azino-bis (3-ethylbenzothiazoline-6-sulfonic acid) (ABTS), Mayer's Hematoxylin Solution, Murashige and Skoog (MS) plant salt mixture, and gellan gum from Wako Pure Chemical Industries, Ltd. (Osaka, Japan); Vero cells (an African Green Monkey kidney-derived cell line) from the American Type Culture Collection (Manassas, VA, USA); fetal bovine serum (FBS) from Hyclone (South Logan, UT, USA); a Cell Counting Kit-8 from DOJINDO (Kumamoto, Japan); 4,6-Diamidino-2-phenylindole Dihydrochloride (DAPI) from Nacalai Tesque (Kyoto, Japan); a Pierce™ BCA Protein Assay Kit, HiMark™ Pre-Stained Protein Standards, MagicMark™ XP Western Protein Standards, SuperSignal™ West Pico PLUS Chemiluminescent Substrate, Medium 199 and Alexa594-goat anti-rabbit IgG from Thermo Fisher Scientific (Waltham, MA, USA); goat anti-mouse κ, horseradish peroxidase (HRP)-goat anti-mouse IgA, HRP-goat anti-mouse IgG, HRP-donkey anti-goat IgG and HRP-goat anti-rabbit IgG from Southern Biotech (Birmingham, AL, USA); rabbit anti-mouse J chain from Proteintech (Wuhan, China); rabbit anti-human/mouse activated-caspase 3 from BD Biosciences (Franklin Lakes, NJ, USA); and rabbit anti-mouse MUC2 from Santa Cruz Biotechnology (Santa Cruz, CA, USA).

### Mice

Female BALB/c mice were purchased from SLC Japan (Shizuoka, Japan) and used at 5–8 weeks of age. Animal care and experiments were performed in accordance with the Act on Welfare and Management of Animals, with the guidelines for the care and use of laboratory animals published by the Ministry of Education, Culture, Sports, Science and Technology of Japan, and with those of the University of Shizuoka. All procedures and protocols were reviewed by the Institutional Animal Care and Use Committee of the University of Shizuoka and were approved by the President of the University of Shizuoka (Approval numbers: 186313, 206,454). The study was reported in accordance with ARRIVE guidelines.

### Construction of a recombinant IgA expression vector

The cDNAs of Stx1-specific IgA heavy, light and J chains were codon-optimized in *Arabidopsis thaliana* and lettuce (*Lactuca sativa*), and then cloned into a cloning vector, pUC57, using GenScript (Piscataway, NJ, USA) to produce each construct (pUC57/opHc, pUC57/opLc, pUC57/opJc). A sequence corresponding to the endoplasmic reticulum retention signal peptide, KDEL (Lys-Asp-Glu-Leu), was also added to the cDNA at the C-terminus of each protein. pUC57/opJc was digested with *Nde*I/*Sac*I. The resulting DNA fragment, opJc cDNA, was ligated into the *Nde*I/*Sac*I-site of pRI201-AN with the DNA Ligation Kit <LONG> , resulting in the production of pRI201/opJc. pGEM5zf/T_*LHCB1.1*_ was digested with *Hin*dIII, and then the cohesive end of *Hin*dIII was blunt-ended using the Klenow fragment. The resulting DNA fragment was digested with *Not*I to obtain the DNA fragment T_*LHCB1.1*_. The T_*LHCB1.1*_ DNA fragment was ligated with *Not*I/*Sma*I-digested pRI201/opJc, resulting in the production of pRI201/opJc-T_*LHCB1.1*_. pUC57/opHc was digested with *Sac*II/*Not*I, in that order, to obtain the cDNA of opHc. The cDNA of opHc was ligated into the *Sac*II/*Not*I site of pGEM5zf/T_*LHCB1.3*_ with the DNA Ligation Kit <Mighty Mix>. The resulting plasmid, pGEM5zf/opHc-T_*LHCB1.3*_, was digested with *Sac*II/*Nsi*I to obtain a DNA fragment, Hc-T_*LHCB1.3*_, which was then ligated into the *Sac*II/*Nsi*I site of pUC57/opLc, resulting in the production of pUC57/opLc-opHc-T_*LHCB1.3*_. The DNA fragment of *Sac*II-digested P_*LHCB*_ was obtained from pGEM5zf/P_*LHCB*_ and inserted into the *Sac*II site of pUC57/opLc-opHc-T_*LHCB1.3*_. The resulting plasmid, pUC57/opLc-P_*LHCB*_-opHc-T_*LHCB1.3*_, was digested with *Kpn*I/*Sal*I. The resulting DNA fragment, opLc-P_*LHCB*_-opHc-T_*LHCB1.3*_, was ligated with *Kpn*I/*Sal*I-digested pRI201/opJc-T_*LHCB1.1*_, resulting in the production of pRI201/dimeric IgA.

### Transformation of *Arabidopsis thaliana*

Transformants of *A. thaliana* plants were generated by means of the floral dip method as previously described^20^. A binary vector, pRI201/dimeric IgA, was introduced into *A. tumefaciens* strain GV3101 by electroporation using a Gene pulsar II (Bio-Rad, Hercules, CA, USA). The resulting *A. tumefaciens* was used to infect *A. thaliana* to produce transgenic plants expressing Stx1-specific IgA. All experiments using plant material complied with the national law (Law Concerning the Conservation and Sustainable Use of Biological Diversity through Regulations on the Use of Living Modified Organisms) and were approved by the internal review committee (Approval number: 618–2103). The recombinant plants were grown within the physical containment level 1-plant (P1P) facility of the University of Shizuoka.

### Protein extraction and concentration

Transgenic plants were cultivated on solidified MS medium with gellan gum containing 50 mg/L of kanamycin in growth chamber, BioTRON NC220 (NK system, Osaka, Japan) at 20 °C, continuous white fluorescent light for 4 weeks. Transgenic plant leaves were ground in liquid nitrogen and leaf proteins were extracted in a protein extraction buffer (50 mM acetate (pH 5.0), 0.5 M NaCl, 0.5 mM EDTA and a protease inhibitor cocktail). Extracted leaf proteins were concentrated by 50%-saturated ammonium sulfate precipitation (AmS) as previously described^19^, followed by ultrafiltration using a Vivaspin 500 (100,000 MWCO, Sartorius, Goettingen, Germany).

### ELISA

The total IgA and IgG concentrations in samples were determined by means of sandwich ELISA as described previously^19,29^. For the IgA concentrations of leaf extracts, goat anti-mouse κ (1 μg/mL) was used as a capture antibody, and HRP-goat anti-mouse IgA (1:1000 dilution) was used as a detection antibody. The IgA concentration was calculated from a standard curve generated with a commercially available mouse IgA myeloma protein, TEPC 15. For the IgG1 concentrations of culture supernatants, goat anti-mouse κ (1:1000) was used as a capture antibody, and HRP-goat anti-mouse IgG (1:1000) was used as a detection antibody. Mouse IgG1 myeloma MOPC 21 was used as a standard. The antigen recognition by antibodies was assessed by means of ELISA using immobilized Stx1B as an antigen^19^. Goat anti-mouse κ (1 μg/mL) plus HRP-donkey anti-goat IgG (1:1000 dilution), or HRP-goat anti-mouse IgA (1:1,000 dilution) were used as detection antibodies. ABTS was used as an HRP substrate. The optical density at 405 nm was read with a microplate reader, SUNRISE Rainbow RC-R (Tecan, Salzburg, Austria).

### SDS-PAGE and immunoblotting

IgA proteins in the leaf extracts were separated by SDS-PAGE using a Mini-PROTEAN TXG Gel (Bio-Rad, Hercules, CA, USA) under reducing (12% gel) and non-reducing (4–15% gel) conditions. Immunoblotting was performed as previously described^14^. As detection antibodies, HRP-goat anti-mouse IgA (1:1000 dilution) was used for detection of the IgA heavy chain, goat anti-mouse κ (1 μg/mL) plus HRP-donkey anti-goat IgG (1:1000 dilution) for the light chain, and rabbit anti-mouse J chain (0.4 μg/mL) plus HRP-goat anti-rabbit IgG (1:5000 dilution) for the J chain. For visualization, an HRP was reacted with SuperSignal™ West Pico PLUS Chemiluminescent Substrate and the chemiluminescent signals were detected with a LAS-3000mini luminescent image analyzer (FUJIFILM, Tokyo, Japan). HiMark™ Pre-Stained High Molecular Weight Protein Standards and MagicMark™ XP Western Protein Standards were used as molecular weight markers.

### Live cell measurement

The neutralization activity of the IgA plantibody was assessed by means of the WST-8 based live cell measurement assay described previously^19^. Vero cells were seeded at 1 × 10^4^ cells/well on a 96-well cell culture plate and then cultured overnight in 10% FBS-containing Medium 199 at 37 °C under a humidified atmosphere of 5% CO_2_/95% air. Vero cells were exposed to an aliquot of the Stx1/antibody mixture that had been prepared by pre-treatment of 100 pg/mL of Stx1 with a leaf extract of a transgenic plant or a culture supernatant of a D11C6 hybridoma for 1 h. After 45-h culture, supernatants were removed, and then living cells were detected by means of WST-8 assaying using a Cell Counting Kit-8 following the manufacturer’s instructions. After incubation for 1.5 h with the WST-8 reagent, the optical density at 450 nm (OD450) was read with a microplate reader, SUNRISE Rainbow RC-R. To determine the live cell number remaining after Stx1 exposure, the amount of formazan product (OD450) in each culture was compared with that without toxin exposure (control).

### Intrarectal administration of Stx1

PBS (total 100 μL) containing 0.1–1.0 µg/mL (except Fig. 3; 5.0 μg/mL) of Stx1 holotoxin was introduced intrarectally into mice using a plastic feeding tube (20G, 38 mm; Instech Laboratories, Plymouth Meeting, PA, USA) under anesthesia by i.p. injection of ketamine/xylazine cocktail (100 mg/kg of ketamine and 10 mg/kg of xylazine). Sixteen hours later, mice were sacrificed by cervical dislocation, and the distal colon (more than 5 cm below the cecum) was collected and fixed in Methanol-Carnoy’s fixative (60% methanol, 30% chloroform and 10% acetic acid) or 10% formalin-PBS. Methanol-Carnoy’s-fixed colons were embedded in paraffin and formalin-fixed colons were embedded in O.C.T. compound (Sakura Finetek, Tokyo, Japan). For electron microscopic analysis, collected colon samples were opened longitudinally, and fixed in 2.5% glutaraldehyde-0.1 M phosphate buffer (PB) and then postfixed in 1% osmium tetroxide (OsO4)-0.1 M PB. For transmission electron microscopy (TEM), they were dehydrated through a graded series of ethanol and embedded in Epon 812. Ultrathin sections were cut, stained with uranyl acetate and lead citrate, and observed under a transmission electron microscope (JEM-1010C; JEOL, Tokyo, Japan). For scanning electron microscopy (SEM), after dehydration, the specimens were critical-point-dried and coated with gold using a sputter coater, and then observed under a scanning electron microscope (JSM- 5600 LV SEM, JEOL).

### Histological analysis

For Alcian blue staining, 4 μm paraffin sections were obtained with a microtome RX-860 (Yamato Koki, Tokyo, Japan). Paraffin sections were dewaxed and hydrated. Sections were fixed with 0.5% (v/v) glutaraldehyde in PBS for 15 min. The fixed sections were stained with 1% Alcian blue in 3% (v/v) acetic acid for 10 min and then nuclei were counterstained with Mayer’s hematoxylin. For immunostaining, 10-µm frozen sections were obtained with a cryostat Leica CM3050 S (Leica Biosystems, Wetzlar, Germany). The sliced sections were blocked with 3% BSA in PBS for 10 min at room temperature. Sections were stained with rabbit anti-mouse activated-caspase 3 (2 μg/mL) plus Alexa594-goat anti-rabbit IgG (5 μg/mL) or rabbit anti-mouse MUC2 (2 μg/mL) plus Alexa594-goat anti-rabbit IgG (5 μg/mL). Nuclei were stained with DAPI (0.1 μg/mL). Immunostaining images were obtained with a fluorescence microscope BZ-810 (KEYECE, Osaka, Japan). The percentage of the MUC2-stained area divided by the total area of the mucous epithelium was determined by using software in the microscope.

### Statistical analysis

Multiple comparisons were made using the Tukey test by means of statistical analysis software, GraphPad Prism 7 (GraphPad Software, San Diego, CA, USA).

## Supplementary Information

Supplementary Figure S1.

## Data Availability

All data generated or analyzed during this study are included in this published article. As a supplementary data, Fig. S1 is uploaded as an unprocessed original version of the immunoblot images for Fig. 1b–g.
