# Supplementary material for: Prevention of Shiga toxin 1-caused colon injury by plant-derived recombinant IgA
Source: Sci Rep. 2022 Oct 26;12:17999. doi: 10.1038/s41598-022-22851-4 (PMC9606113; doi:10.1038/s41598-022-22851-4)
Supplement: Supplementary file 1 — Supplementary Figure S1. [file 41598_2022_22851_MOESM1_ESM.pdf]

SUPPLEMENTARY DATA

Prevention of Shiga Toxin 1-caused Colon Injury by Plant-derived Recombinant IgA

Katsuhiro Nakanishi [1], Taichi Takase [1], Yuya Ohira [1], Ryota Ida [1], Noriko Mogi [1], Yuki Kikuchi [1], Minami Matsuda [1], Kohta Kurohane [1], Yoshihiro Akimoto [2], Junri Hayakawa [3], Hayato Kawakami [2], Yasuo Niwa [4], Hirokazu Kobayashi [4], Eiji Umemoto [1], Yasuyuki Imai [1, *]

[1] Laboratory of Microbiology and Immunology, School of Pharmaceutical Sciences, University of Shizuoka, Shizuoka City, Shizuoka 422-8526, Japan

[2] Department of Anatomy, Kyorin University School of Medicine, Mitaka, Tokyo 181-8611, Japan

[3] Laboratory for Electron Microscopy, Kyorin University School of Medicine, Mitaka, Tokyo 181-8611, Japan.

[4] Laboratory of Plant Molecular Improvement, Graduate Division of Nutritional and Environmental Sciences, University of Shizuoka, Shizuoka City, Shizuoka 422-8526, Japan

***Correspondence:** Yasuyuki Imai, Laboratory of Microbiology and Immunology, School of Pharmaceutical Sciences, University of Shizuoka, Shizuoka City, Shizuoka 422-8526, Japan.

Tel: +81-54-264-5120; fax: +81-54-264-5099

E-mail: imai@u-shizuoka-ken.ac.jp

Figure 1b

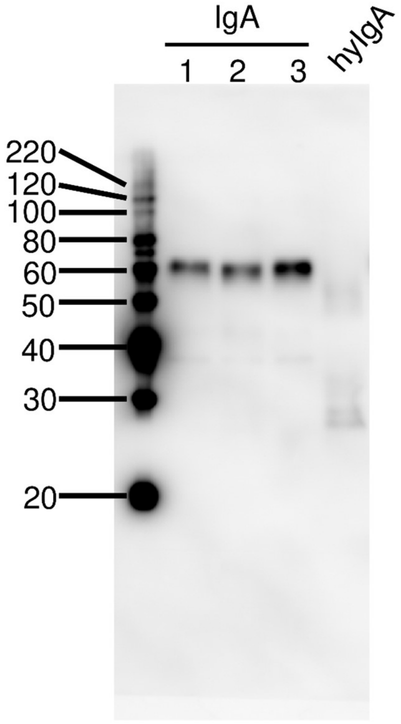

Figure 1c

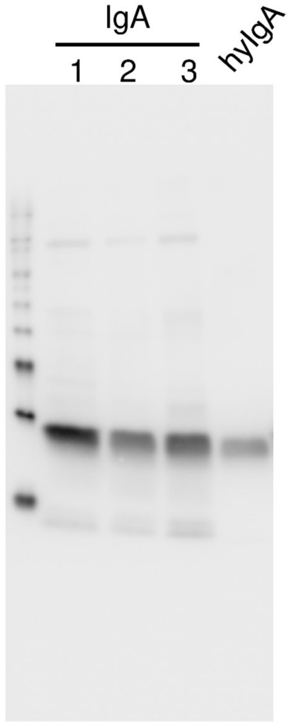

Figure 1d

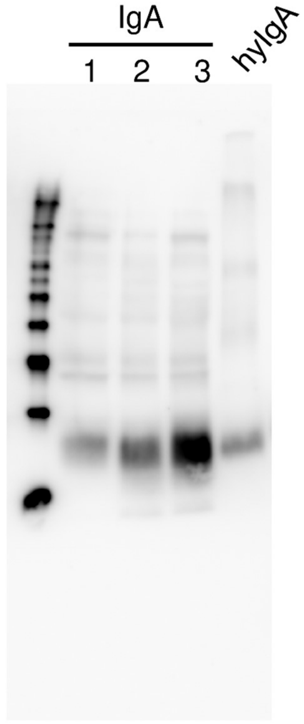

Figure 1e

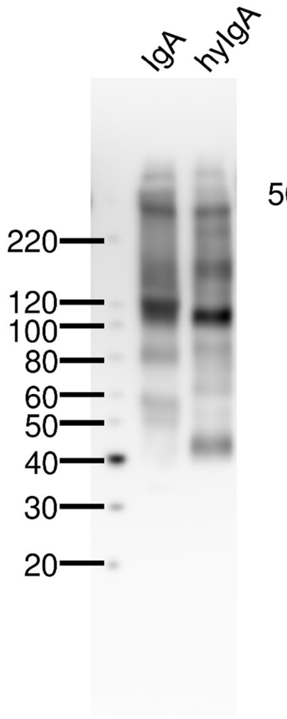

Figure 1f

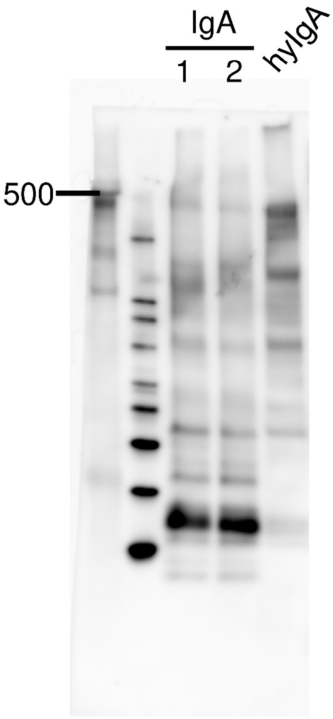

Figure 1g

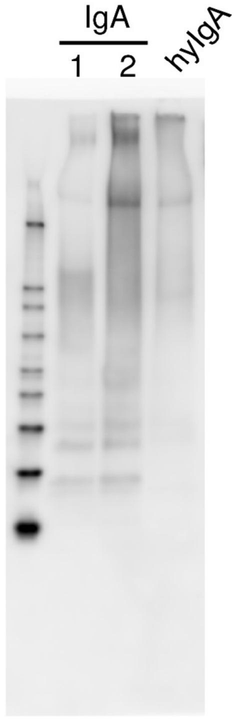

Supplementary Figure S1. Original full-length blot images corresponding to the Figure 1-bcdefg. Lanes appear under the labels of hyIgA correspond to the data of Figure 1. Lanes appear under the labels of IgA, the lane 3 (panels Figure 1b, 1c and 1d) and the lane 2 (panels Figure 1f and 1g) correspond to the data of Figure 1. Lanes of samples simultaneously applied in the same gel (lanes 1 and 2 in the panels Figure 1b, 1c and 1d; lane 1 in the panels Figure 1f and 1g) are not related to this study.
